# Comparison of the therapeutic effects of the dietary and topical forms of Zizyphus jujuba extract on oral mucositis induced by 5-fluorouracil: A golden hamster model

**DOI:** 10.4317/jced.52198

**Published:** 2015-04-01

**Authors:** Omid Koohi-Hosseinabadi, Azadeh Andisheh-Tadbir, Parisa Bahadori, Masood Sepehrimanesh, Maryam Mardani, Nader Tanideh

**Affiliations:** 1 Laboratory Animals Center, Shiraz University of Medical Sciences, Shiraz, Iran; 2 Department of Oral and Maxillofacial Pathology, School of Dentistry, Shiraz University of Medical Sciences, Shiraz, Iran; 3 Students’ Research Committee, School of Dentistry, International Branch, Shiraz University of Medical Sciences, Shiraz, Iran; 4 Gastroenterohepatology Research Center, Shiraz University of Medical Sciences, Shiraz, Iran; 5 Department of Oral Medicine, School of Dentistry, Shiraz University of Medical Sciences, Shiraz, Iran; 6 Stem Cell and Transgenic Technology Research Center and Department of Pharmacology, School of Medicine, Shiraz University of Medical Sciences, Shiraz, Iran

## Abstract

**Background:**

Oral mucositis (OM) is a common inflammatory complication among cancerous patients as an adverse effect of chemotherapy and radiotherapy. The aim of this study was to evaluate the effects and identify the appropriate route of administration of extract of Zizyphus jujuba in 5-flurouracile induced OM induction in golden hamster.

**Material and Methods:**

In a prospective randomized double blind animal study, OM was induced in 56 male golden hamsters by 5-FU (60 mg/kg) on days 0, 5, and 10 of the study. The cheek pouch was scratched with a sterile needle on once daily on days 3 and 4. On days 14-17, 300 mg/kg dietary and 20% Z. jujuba gel and gel base groups were treated and then compared with a control group. Microscopic scores and pouch content of malondialdehyde (MDA), plus activities of superoxide dismutase and myeloperoxidase in pouch tissue were evaluated.

**Results:**

Histopathology scores of mucositis were lower in the systemic and 20% Z. jujuba gel groups than the gel base and control groups (P<0.05). Also, lower MDA level and higher activities of MPO and SOD were detected in the systemic and 20% Z. jujuba gel groups in comparison to the gel base and control groups (P<0.001).

**Conclusions:**

The use of Z. jujuba hydroalcoholic extract in systemic and topical forms may be associated with reduced intensity of OM, diminished concentration of MDA, and increased activity of MPO and SOD on induced OM in golden hamster undergoing 5-FU consumption.

** Key words:**Oral mucositis, 5-flurouracil, Zizyphus jujube, oxidative stress, histopathology score.

## Introduction

Inflammation is a physiological response of body especially vascular tissues to harmful stimuli, including infections and tissue injury. As the primary interface between the body and the external environment, the skin provides the first line of defense against traumatic injury and invasion by microbial pathogens (1). On the other hand, for orally digested chemical agents and microbiota, oral mucosa is the first line of defense and approximately in all cases, it suffer histopathological and inflammatory injuries. Oral mucositis (OM) was first described in 1980s and is the painful inflammation and ulceration of the mucous membranes lining the oral cavity, usually as an adverse effect of treatment for cancer (2). Both chemotherapy and radiation therapy can interfere with maturity, cellular growth, and renewal rate of basal epithelial cells, causing changes to normal turnover and cell death (3). Indeed, OM is the result of a series of inflammatory changes in the epithelial and subepithelial cells of the oral mucosa and is a debilitating toxicity of chemoradiotherapy for head and neck cancer (HNC) (4,5). HNC, principally squamous cell carcinoma, is one of the main oncological problems owing to its high mortality rate and the side-effects of the treatment (5). One of the chemotherapeutic drugs that can be induced OM is fluorouracil (5-FU). 5-FU, which trademarked as Adrucil®, Carac® and Efudex®, is a pyrimidine analog drug which is used in the treatment of cancer (6). 5-FU has been administered systemically for anal, breast, colorectal, esophageal, stomach, pancreatic and skin cancers (especially HNC) and topically for actinic keratoses and Bowen’s disease (3).

Management of OM via topical approaches should address efficacy, patient acceptance, and appropriate dosing. Although, we conducted some reports about using dexamethasone as chemical drugs for prevention of OM (7,8), but medicinal plants have attracted more attention. A large number of plant species contain various bioactive compounds exhibiting health beneficial properties, anti-oxidative, anti-inflammatory and mainly antimicrobial effects, and their preventive and therapeutic use increases (1). In our point of view, it has been reported that Aloe vera mouthwash may reduce radiation-induced OM in HNC patients (9). Also, our previous studies demonstrated that some medicinal plants such as bilberry (10), St. John’s wort (2) and Marigold (3) could effectively intervene against development of OM.

Zizyphus jujuba is a thorny rhamnaceous plant that is widely distributed in Europe and Southeastern Asia (11). Pharmacologically active peptides including cyclopeptide alkaloids, flavonoids, sterols, jujuboside A, jujuboside B, lauric acid, and triterpenoid saponins have been isolated and chemically identified from different species of *Z. jujuba* (12). Fruits of this plant are edible and different parts of *Z. jujuba* possess multiple medicinal properties such as antifertility, analgesic, and antidiabetes (11). It also has been used as tranquilizer, analgesic, and anticonvulsant in folk medicine in India (13). However, there are no scientific studies about the effect of *Z. jujuba* fruit in prevention and treating of OM. Considering the debilitating conditions that OM induced by chemoradiotherapy can cause in cancer patients and the lack of a definite treatment strategy, and based on the data implicating the anti-inflammatory, analgesic and wound healing activities of *Z. jujube*, we decided to evaluate and compare the effects of hydroalcoholic extract of the fruit of *Z. jujuba* in two forms, topical and dietary, on 5-FU induced OM in golden hamster.

## Material and Methods

-Animals and housing

This study was approved by the Research and Ethics Committee of Shiraz University of Medical Sciences, and all relevant considerations about animal rights were taken into this study. This randomized animal trial was performed in the Laboratory Animal Center of Shiraz University of Medical Sciences. Fifty six male golden hamsters (100 to 120 g, 6-8 weeks old) were taken from Laboratory Animal Center of Shiraz University of Medical Sciences, Shiraz, Iran and were housed in standard cages under a 12-h light cycle (lights on at 7:00 pm) with an ambient temperature of 22 ± 2°C, and 55% relative humidity. Hamsters were given a 3-day acclimation period with an access to normal chow and water ad libitum before the experiment. During their stay in the respective housing conditions, they were removed from their cages every 3 days for cleaning the cages and renewing their food and water supply. During the experiment, access to standard laboratory diet and water was allowed ad libitum.

-Preparation of hydroalcoholic extract

The fruit of *Z. jujube* was collected from Shiraz in the Fars province of Iran. The fruits were dried at room temperature and powdered in a grinder. Aqueous ethanol (75%) was added to the powdered materials (500 g), and stirred for one hour. The mixture was kept at room temperature for 48 hours. Following filtration, ethanol was evaporated under reduced pressure at 40ºC. The remained water extract was dried at oven temperature of 50ºC. Finally, 20% *Z. jujube* hydroalcoholic extract was prepared in pharmacy laboratory.

-Chemotherapy induced OM

Fifty six adult male golden hamsters were randomly allocated into four groups (14 hamsters in each group). All animals received three intraperitoneal injections of 5-FU on days 0, 5, and 10 at a dose of 60 mg/kg with insulin needles following the protocol proposed by Sonis *et al.* (14). To mimic the clinical effect of chronic irritation, the cheek pouch mucosa was scratched superficially with the tip of an 18-gauge sterile needle after anesthetizing the area. Two horizontal linear scratches were made across the everted cheek pouch once daily on days 3 and 4. The treatment was started on day 12 and 1st group of hamsters served as controls and didn’t receive any treatment; group 2 received gel base used in preparation of the topical gel; group 3 were treated with topical *Z. jujuba* extract gel 20% on the cheek pouch mucosa of both sides once a day (morning) and the affected area was completely covered. Group 4 were fed *Z. jujuba* hydroalcoholic extract with dose 300 mg/kg once a day (morning) in dietary root during study. To make sure that it was not swallowed, animals weren’t allowed to eat or drink for 30 minutes after the drug applications. Animals were weighed daily. Seven hamsters from each group were randomly selected for killing on days 14, and 17. At the time of sacrifice, they were anesthetized with 100 mg/kg ketamine 10% (Alfasan, Netherlands) and 10 mg/kg xylasine 2% (Alfasan, Netherlands).

-Histopathological study

The both side cheek pouch tissue of hamsters in all groups were harvested on day 14 and 17. Tissue obtained from the right side pouch was proceed and stained with hematoxylin-eosin according to previous reported procedure (15-17). The specimens were examined histopathologically and received scores of 0 to 3, as follows (18).

- Score 0, normal epithelium and connective tissue without vasodilatation; absent or mild inflammatory infiltrate; absence of bleeding, ulcers and abscesses

- Score 1, mild vascular hyperemia; areas of reepithelialization; mild inflammatory infiltrate with a prevalence of mononuclear cells; no hemorrhagic areas, ulcerations or abscesses

- Score 2, moderate vascular redness; areas of epithelial degeneration; inflammatory infiltration with prevalence of neutrophils; hemorrhagic areas, edema and occasional ulcerations; absence of abscesses

- Score 3, severe hyperemia and vascular vasodilatation; inflammatory infiltration

-Measurement of malondialdehyde (MDA)

Tissues obtained from the left side pouch were stored under liquid nitrogen until measurment. Tissue malondialdehyde (MDA) assessment was performed via the measurement of thiobarbituric acid reactive substances (TBARS) in PBS tissue homogenate (19), because MDA is one of the end-products of lipid peroxidation (LPO), and the extent of LPO is most frequently measured by estimating MDA levels (20).

-Superoxide dismutase (SOD) assay

Total SOD activity was evaluated with SOD detection kit (BiorexFars, Iran) according to the manufacturer’s instructions. The role of SOD is to accelerate the dismutation of the toxic superoxide (O2−) produced during oxidative energy processes to hydrogen peroxide and molecular oxygen. This method employs xanthine and xanthine oxidase (XOD) to generate superoxide radicals which react with 2-(4-iodophenyl)-3-(4-nitrophenol)-5-phenyltetrazolium chloride (INT) to form a red formazan dye. The SOD activity is then measured by degree of inhibition of this reaction. One unit of SOD is that which causes 50% inhibition of the rate of reduction of INT under the conditions of the assay. SOD levels were recorded at 505 nm and through a standard curve and expressed as U/g wet tissue.

-Myeloperoxidase (MPO) assay

MPO activity can be measured in tissues by assays using hydrogen peroxide and odianisidine dihydrochloride as substrates (21). Briefly, cheek pouch tissues which collected on days 14 and 17, were weighed and homogenized in a solution containing 0.5% (w/v) hexadecyltrimethylammonium bromide dissolved in 10 mmol/L potassium phosphate buffer (pH 7.4) and centrifuged for 30 minutes at 20,000 g at 4°C. An aliquot of supernatant was then removed and added to a reaction mixture containing 1.6 mmol/L tetramethylbenzidine and 0.1 mmol/L hydrogen peroxide (H2O2). The rate of change in absorbance was measured spectrophotometrically at 650 nm. MPO activity was defined as the quantity of enzyme required to degrade 1 mmol of H2O2 at 37°C and was expressed in U/g wet tissue.

-Statistical analysis

All statistical analyses were carried out using SPSS® statistical software, version 20.0 (SPSS Inc., Chicago, IL, USA) for Windows®. Data are expressed as the mean ± standard deviation (SD) from all independent experiments. One-Sample-Kolmogorov-Smirnov test was used to test for normality of distribution of the collected data. A two tailed paired sample T tests were used to compare MDA level, SOD and MPO activities and pathology score between days 14 and 17 in control group and treatment groups. Differences were considered significant when *P*<0.05.

## Results

-Histopathology evaluation

**Figure 1:**
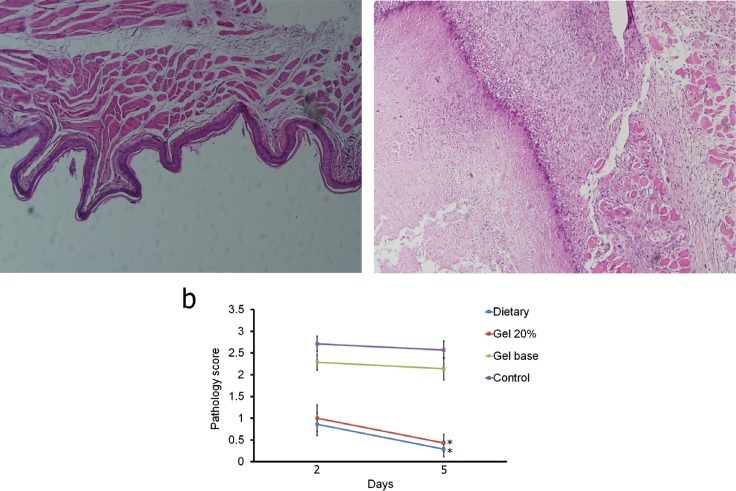
The histopathology lesions a) and score b) comparison between four different groups in two days of sampling. In histopathological slides, left is score 0 and right is score 3. Significant differences (P<0.05) between two days were indicated by asterisk.

The maximum severity of OM was seen on day 12 (base on clinical examination) and the treatment thus was started on this day. There is no histopathologic score (HPS) difference between the gel base and control groups and between the topical gel and systemic treatment groups on both days. Also, only significant decreases are seen after 5 days treatment in comparison to 2 days treatment in the HPS in response to topical gel and systemic treatment groups (*P*<0.05). These declines indicating less severe inflammation in the topical gel and systemic treatment groups (Fig. 1). Epithelialization and wound healing were detected in the topical gel and systemic treatment groups, whereas extensive infiltration of inflammatory cells, hemorrhage and ulcer were observed in the control and gel base groups. On days 12 and 17, HPS for the topical gel and systemic treatment groups were less than gel base group (*P*<0.001). Also on these two days, HPS for the topical gel and systemic treatment groups were less than that for the control group (*P*<0.001).

-Tissue MDA level

**Figure 2:**
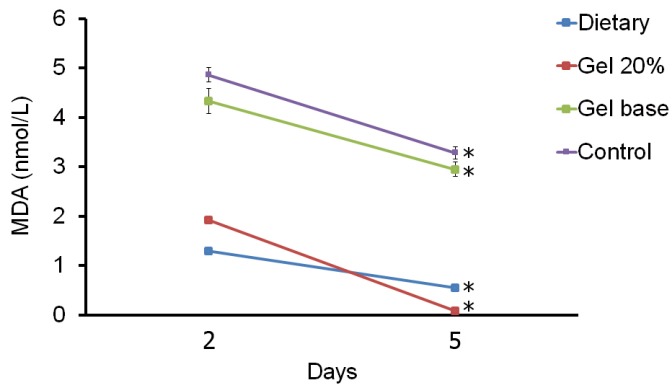
The mean ± SD comparison of malondialdehyde (MDA) concentration between four different groups in two days of sampling. Significant differences (P<0.05) between two days were indicated by asterisk.

Tissue MDA concentration in experimental groups on 2 and 5 days after onset of treatment are shown in figure 2. There are significant differences between MDA concentrations in all four groups between days 2 and 5. Also, MDA concentration is higher in the control and gel base groups than in the topical gel and systemic treatment groups on all days (*P*<0.001). Although, MDA concentration of the topical gel was higher than the systemic treatment group on day 14 (*P*=0.026), but on day 17, MDA level in the topical gel was lower than the systemic treatment group (*P*=0.728). Totally, both systemic and topical treatment could diminish the MDA level obviously.

-Tissue MPO activity 

**Figure 3:**
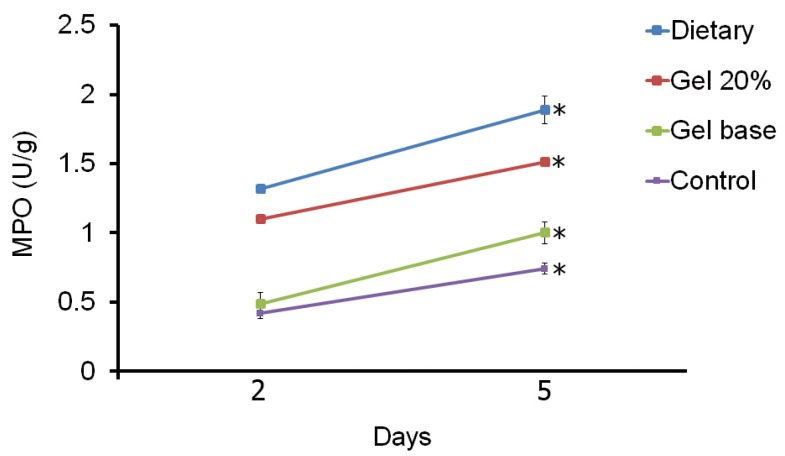
The mean ± SD comparison of myeloperoxidase (MPO) acti-vity between four different groups in two days of sampling. Significant differences (P<0.05) between two days were indicated by asterisk.

Tissue MPO activity in experimental groups on 2 and 5 days after onset of treatment are shown in figure 3. There are significant differences between MPO activity in all four groups between days 2 and 5. Also, MPO had more activity in the systemic treatment and the topical gel groups than in the control and gel base groups on all days (*P*<0.001). Difference between the control and gel base groups and also between the systemic treatment and the topical gel groups on day 14 (2 days after treatment) were not significant (*P*=0.987 and *P*=0.180, respectively). On day 17 (5 days after treatment) significant differences were detected between the systemic treatment and the topical gel groups (*P*=0.001), between the systemic treatment and the control and gel base groups (*P*<0.001) and between the topical gel and the control and gel base groups (*P*<0.001). On day 17, there was no significant difference between the control and gel base groups (*P*=0.058).

-Tissue SOD activity 

**Figure 4:**
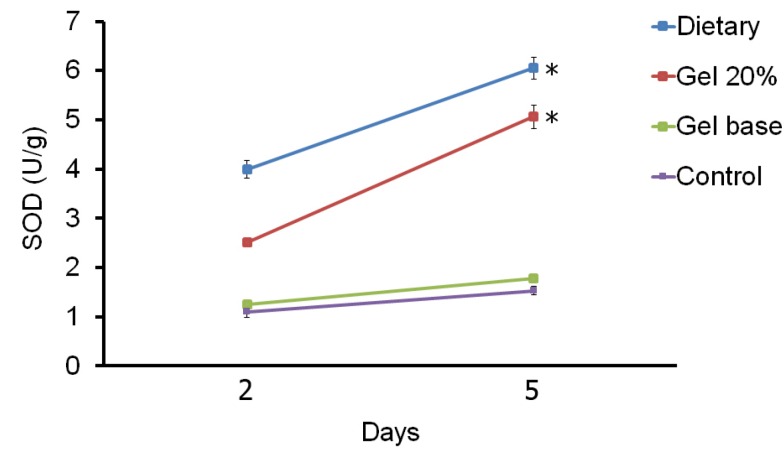
The mean ± SD comparison of superoxide dismutase (SOD) activity between four different groups in two days of sampling. Significant differences (P<0.05) between two days were indicated by asterisk.

Tissue SOD activity in experimental groups on 2 and 5 days after onset of treatment are shown in figure 4. There are significant differences between SOD activity only in the systemic treatment and the topical gel groups between days 2 and 5. SOD had more activity in the systemic treatment and the topical gel groups than the control and gel base groups on all days (*P*<0.001). Although, difference between the control and gel base groups on day 14 (2 days after treatment) was not significant (*P*=0.996), but on this day, the systemic treatment and the topical gel groups showed significant difference (*P*<0.001). On day 17 (5 days after treatment) significant differences were detected between the systemic treatment and the topical gel groups (*P*=0.001), between the systemic treatment and the control and gel base groups (*P*<0.001) and between the topical gel and the control and gel base groups (*P*<0.001). On day 17, there was no significant difference between the control and gel base groups (*P*=0.222).

## Discussion

This study compared the effects of two different drug forms of *Z. jujuba* extract, topical gel 20% and 300 mg/kg dietary forms, on the healing of OM induced by 5-FU in golden hamster. Our results demonstrated that all two drug forms can induce wound healing by decreasing MDA concentration and elevating MPO and SOD activity. Also, the systemic dietary form was approximately more potent than topical gel.

Although, inflammatory diseases are currently treated with steroidal and non-steroidal anti-inflammatory drugs (NSAIDs) (1), but, there are several reports about the healing and anti-inflammatory effects of recombinant proteins and medicinal plants in OM, as an inflammatory disease of oral mucosa, in human and animal models (2-4,10,18,22-25). OM is the most prevalent side effect of the anticancer treatment, accounting for more than 90% of patients receiving radiotherapy to treat HNC (26). In two randomized, placebo-controlled trials conducted in HNC patients undergoing postoperative chemoradiotherapy and in patients receiving definitive chemoradiotherapy for locally advanced HNC, intravenous palifermin, a truncated human recombinant keratinocyte growth factor (KGF), administered weekly for 8 weeks decreased severe OM (4,23). On the point of medicinal plant view, the effect of essential oil from seeds of Z. jujube on 12-O-tetradecanoylphorbol-13-acetate (TPA) induced skin inflammation in experimental mice was investigated by Al-Reza and his colleagues. They reported that *Z. jujuba* essential oil inhibited the inflammatory responses of skin inflammation in animal model. Also, they concluyded that the essential oil of *Z. jujuba* seeds might accelerate the development of new drugs for various inflammatory diseases (1). Also, the use of mouthwash containing 1% Chamomilla recutita extract could be associated with reduced incidence, intensity, and duration of mucositis in adults patients undergoing allogenic hematopoietic stem cell transplantation which suffered from OM (22). In another study, the effectiveness of Matricaria recutita Linn (chamomile) in the prevention and control of radiation-induced OM was evaluated. Their results showed that group with chamomile gel had the faster clinical improvement (24). In all previous reports, plants and their extracts had no prophylactic effect on the onset of OM, but it was proven to be effective in decreasing the severity of this condition. Also, it has been reported that the beneficial effects of these traditional plants may be due to polyphenols content which reviewed by Varoni *et al.* (25).

On the other hand, oxidative injury from free radical generation is involved in the pathophysiology of many human diseases (13). Several investigations have been performed to elucidate the exact role of highlyspecific molecules or mechanisms underlying the OM development (7,18,27). It has been demonstrated that hydroalcoholic extract of *Z. jujube* can reverse oxidative stress induced by pentylenetetrazole (PTZ) and electroshock (13). Our results rendered more evidence to the critical role of free radicals and protective roles of antioxidative enzymes included SOD and MPO in pathogenesis of OM. These protective effects are useful seen in the groups receiving systemic and topical forms of *Z. jujube*.

## Conclusions

According to the findings of present study, *Z. jujube* might be effective agent in treatment of OM. However, further studies must be performed on the finding of its active anti-inflammatory and antioxidative components in different diseases and clinical trials.
